# Artificial Intelligence Approach in Hip Prosthesis Identification and Addressing Radiographic Outcome Measures

**DOI:** 10.1016/j.artd.2025.101717

**Published:** 2025-06-03

**Authors:** Omar Musbahi, Savvas Hadjixenophontos, Saran S. Gill, Iris Soteriou, Kyriacos Pouris, Takuro Ueno, Justin P. Cobb

**Affiliations:** a MSk Lab, White City Campus, Imperial College London, London, UK; b Department of Electrical and Electronic Engineering, Imperial College London, London, UK; c Department of Orthopaedic Surgery, Toyama Prefectural Central Hospital, Toyama, Japan

**Keywords:** Computer vision, Artificial intelligence, Machine learning, Hip, Detection

## Abstract

**Background:**

Radiographic assessment is crucial for the success of a hip arthroplasty procedure as a correctly positioned prosthesis indicates favorable long-term outcomes. This project aims to develop a novel artificial intelligence (AI)–based method that can (1) automatically identify the presence of a hip resurfacing prosthesis in radiographs and (2) calculate the radiographic neck-shaft angle (NSA) of the prosthesis from 2-dimensional plane images using both anterior-posterior (AP) and lateral radiographs with high accuracy.

**Methods:**

Using a computer vision and pattern recognition algorithm, the femur shaft and prosthesis regions were identified, and their respective angles were extracted for NSA calculation. A neural network (NN) was then trained using clinician-generated AP radiograph NSAs as ground truths and AI-generated AP and lateral NSAs as features. Spearman's correlation and Kruskal-Wallis tests were calculated to explore any significant association between the final AI-generated and clinician-generated AP radiographic NSAs. Mean absolute error (MAE) and R-squared values were calculated with and without the NN model to identify the model's accuracy and variability.

**Results:**

There was a statistically significant correlation between the final AI-generated AP radiographic NSAs and the clinician-generated AP radiographic NSAs (r_s_ = 0.93, *P* < .01). MAE, R^2^, and r_s_ without the NN were 3.09, 0.37, and 0.83 (*P* < .01), respectively. MAE and R^2^ with the NN were 1.94 and 0.53, respectively.

**Conclusions:**

This study demonstrates that the identification of hip resurfacing prostheses using AI is feasible. By incorporating additional features such as the lateral NSA, the model can provide an accurate prediction of the AP radiographic NSA, closely approximating the ground truth.

## Introduction

Globally, more than 1 million total hip arthroplasties (THAs) are performed per year, demonstrating significant enhancements in quality of life across all age groups [1]. The primary indication of THA is osteoarthritis, with a study from the Arthritis Program of the Centers of Disease Control and Prevention estimating a lifetime risk of symptomatic hip osteoarthritis being 18.5% and 28.6% in males and females, respectively [2,3]. Despite its widespread use and documented success, the THA may not be the optimal procedure for all patients [[4], [5], [6]]. Younger patients, in particular, may benefit from less invasive alternatives [4,5]. Hip resurfacing arthroplasty (HRA) has gained popularity as one such alternative, offering greater postoperative mobility, improved gait-related outcomes, and a low failure rate [7,8]. As such, there is a need to develop technology that can help with HRA planning, aiming to increase its uptake [[9], [10], [11], [12]].

The planning of a hip arthroplasty involves detailed radiographic analysis to determine parameters such as cup and stem sizes, neck-shaft angle (NSA), and cup depth [[13], [14], [15], [16]]. While 3-dimensional (3D) preoperative HRA planning software has been explored in previous studies, research on computer vision (CV) models for HRA planning remains limited. Studies have highlighted the potential of CV and artificial intelligence (AI) in the detection of pathologies on various imaging modalities, pelvic radiographs, chest computed tomography (CT), and magnetic resonance imaging (MRI) scans [13,[17], [18], [19]]. However, their application specifically for identifying and planning HRA prostheses has not yet been extensively explored.

CV has been effectively employed to identify THA prosthesis and calculate angles such as the hip-knee-ankle angle and NSA, also known as the caput-collum-diaphyseal angle and Sharp’s angle, across various imaging modalities with high levels of accuracy [20,21]. While effective preoperative planning and achieving optimal NSA have shown to improve function in THA, CV for HRA has not been specifically explored [20]. The development of a CV model capable of determining the NSA of hip prostheses from 2-dimensional imaging could serve as an initial screening tool for orthopaedic surgeons [18].

This study aims to develop a novel CV and AI-based method, specific for hip prosthesis detection. Our objectives are to use CV techniques to identify the presence of an HRA prosthesis in radiographs and accurately calculate the radiographic NSA of the prosthesis using both anterior-posterior (AP) and lateral radiographs, with high accuracy.

## Material and methods

### Patient selection

A retrospective study was conducted using a single-center orthopaedic surgery dataset containing consecutive patients who underwent HRA between July 2013 and December 2022.

Inclusion criteria to be added into the database were: Any patient scheduled for, or having already undergone, pelvic or lower limb surgery; subjects with no previous history or current symptoms of lower limb disorders and subjects aged between 18 and 90 years; and those who can ambulate safely without the use of assistive devices. The exclusion criteria included subjects lacking capacity to consent, subjects who do not understand written or verbal English, and subjects with any neurological condition affecting the lower limbs.

In our study, we only reviewed participants who had undergone Birmingham Hip Resurfacing (BHR), with a Smith & Nephew BHR System [22]. Patients who had missing, unavailable, or distorted AP or lateral postoperative pelvic radiographs and patients who underwent THA or H1 HRA were excluded. These were excluded due to the challenges of identifying a stemless component through existing open-source CV programs.

### Ground truth–clinician NSA calculation

**Figure 1:**
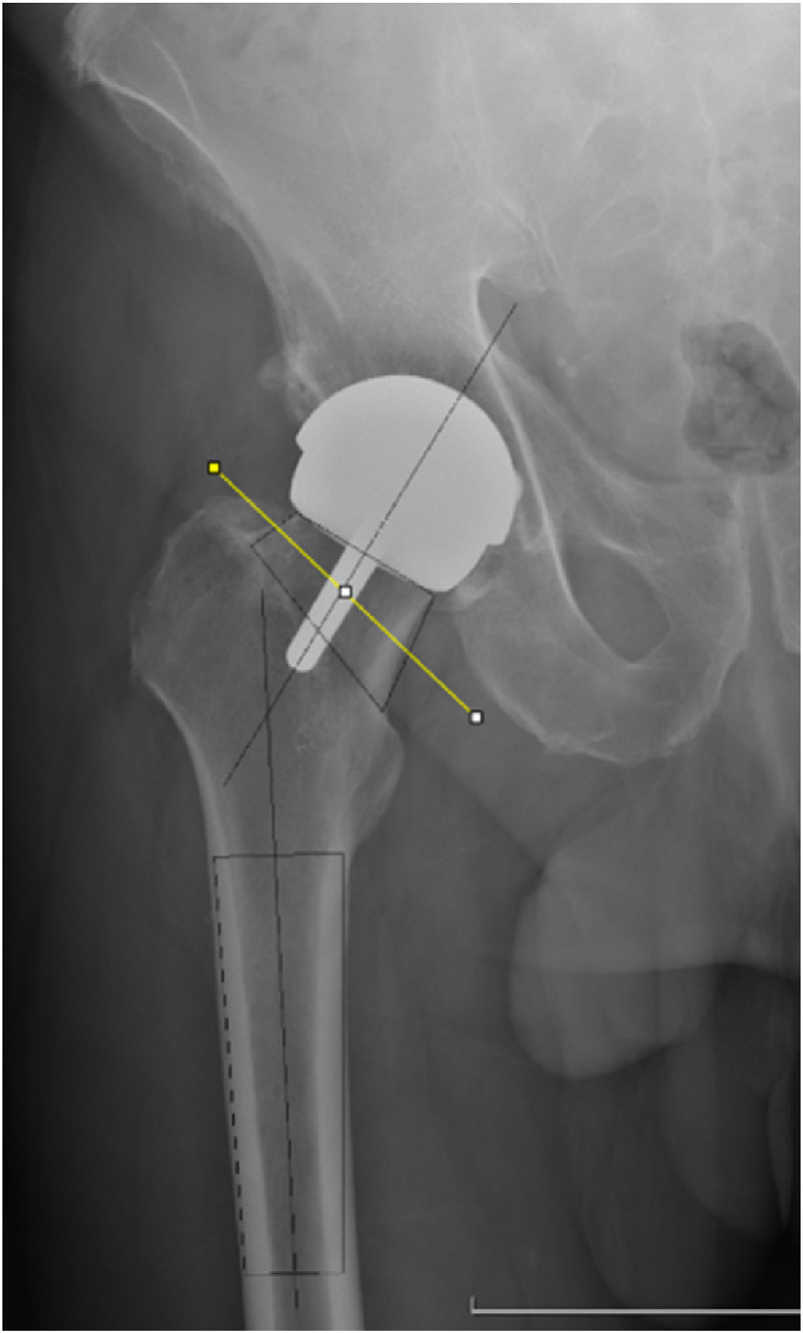
Clinician-generated NSA calculation. NSA, neck-shaft angle.

The NSA angle was calculated using the trapezoid technique for determining the axes, which considers the femoral neck as a quadrangle [23]. The axis of the femoral neck is determined by taking the midpoint of 2 femoral neck segments and by drawing a straight line through these 2 points using ImageJ. The axis of the femoral shaft or of the stem of the hip prosthesis can be determined using the same technique. This is shown in Figure 1.

Figure 1 describes this clinician-generated NSA calculation, using the trapezoid technique to determine the axes by taking the midpoint of 2 segments of specific landmarks, such as the femoral shaft, neck, or stem.

### AI system NSA calculation

#### Preprocessing

**Figure 2:**
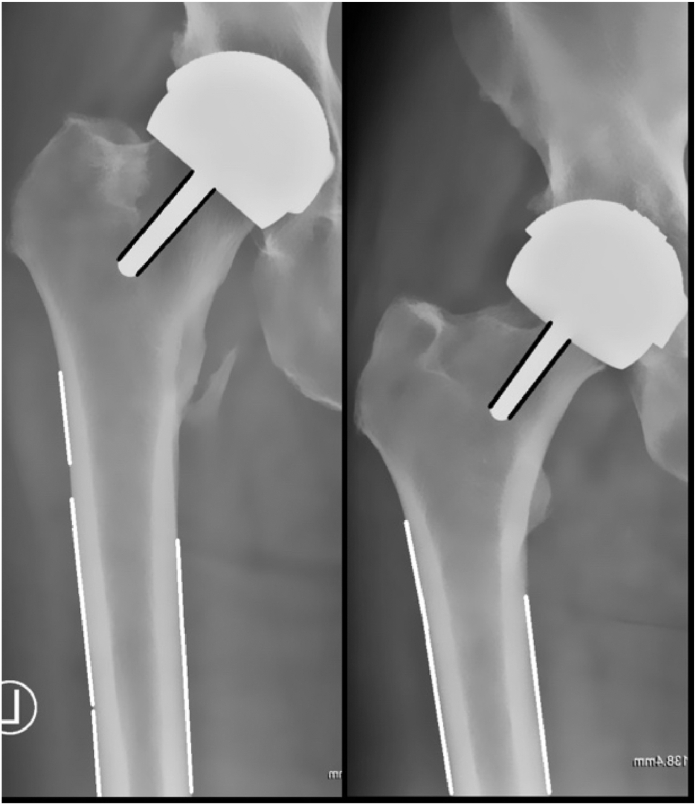
Computer vision algorithm using edge detection to compute NSA. NSA, neck-shaft angle.

Preprocessing involved converting the images to grayscale and by applying a bilateral filter to reduce the noise in the background of the images while preserving significant edges [24]. Subsequently, we used a mean square error (MSE) CV algorithm with 5 reference prostheses images, to automatically identify the coordinates of the prosthesis (Fig. 2). The femur coordinates were assigned as the area under the lower side of the detected prosthesis segments.

Figure 2 shows a visualization of the CV model, created using the edge detection to compute and calculate NSA.

#### Edge detection

The linear edges of the femur and the prosthesis extensions are approximately parallel to the true lines used for the NSA calculation. To capture the angle of the edges, we used the Hough Transform (HT) introduced by Hough et al., an edge detection algorithm [25]. The algorithm fits straight lines through all possible instances of the radiographs and then selects the ones with the highest number of instances as the detected lines. The algorithm then returns an array of all detected lines indicating their radius and angles. The minimum length of detected lines and threshold of instances determining them were fine-tuned in the HT function by inspection using a small portion of the sample size. We used the median of both arrays to ignore any possible outliers caused by the background of the radiographs. The NSA was then computed as the sum of absolute values of the median femur and median prosthesis angles.

#### Reliability measures

To determine if the outputs of the CV algorithm were random, Spearman's correlation (r_s_) was calculated to explore the association between the AI-generated and clinician-generated AP radiographic NSAs. To also ensure reliable results, the entire experiment including processing was repeated 1000 times. For each repetition, the patient order was shuffled to ensure variation in the training and testing data.

#### Processing

**Figure 3:**
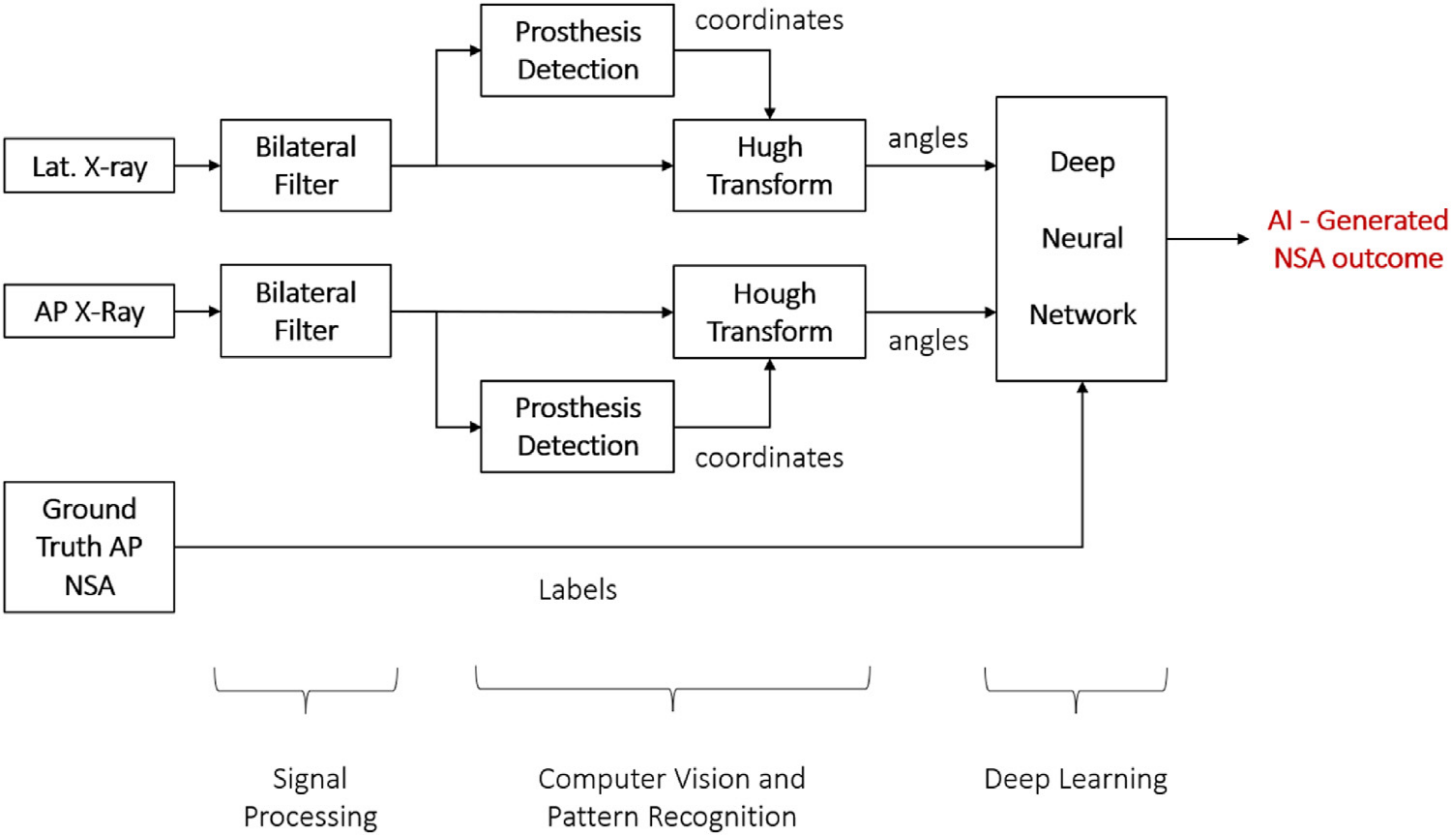
System overview.

The lateral images were also processed by the same CV algorithm producing the lateral NSAs. Both lateral and AP NSAs were then used as features to train a Deep Neural Network (DNN) in producing a corrected value for the AP NSAs. An 80:20 training-testing split was used for the experiment. The DNN model comprised of 2 hidden layers with 128 neurons each. Adam’s optimizer was used, and the loss function was Binary Cross Entropy. Figure 3 overviews the development of the model.

Figure 3 shows the process of developing the CV model. Starting with preprocessing radiographic images using bilateral filtering, detecting prostheses, angles are then extracted using the HT, and finally NSAs are calculated using a DNN. Clinician-calculated NSAs are considered the ground truth.

## Results

**Figure 4:**
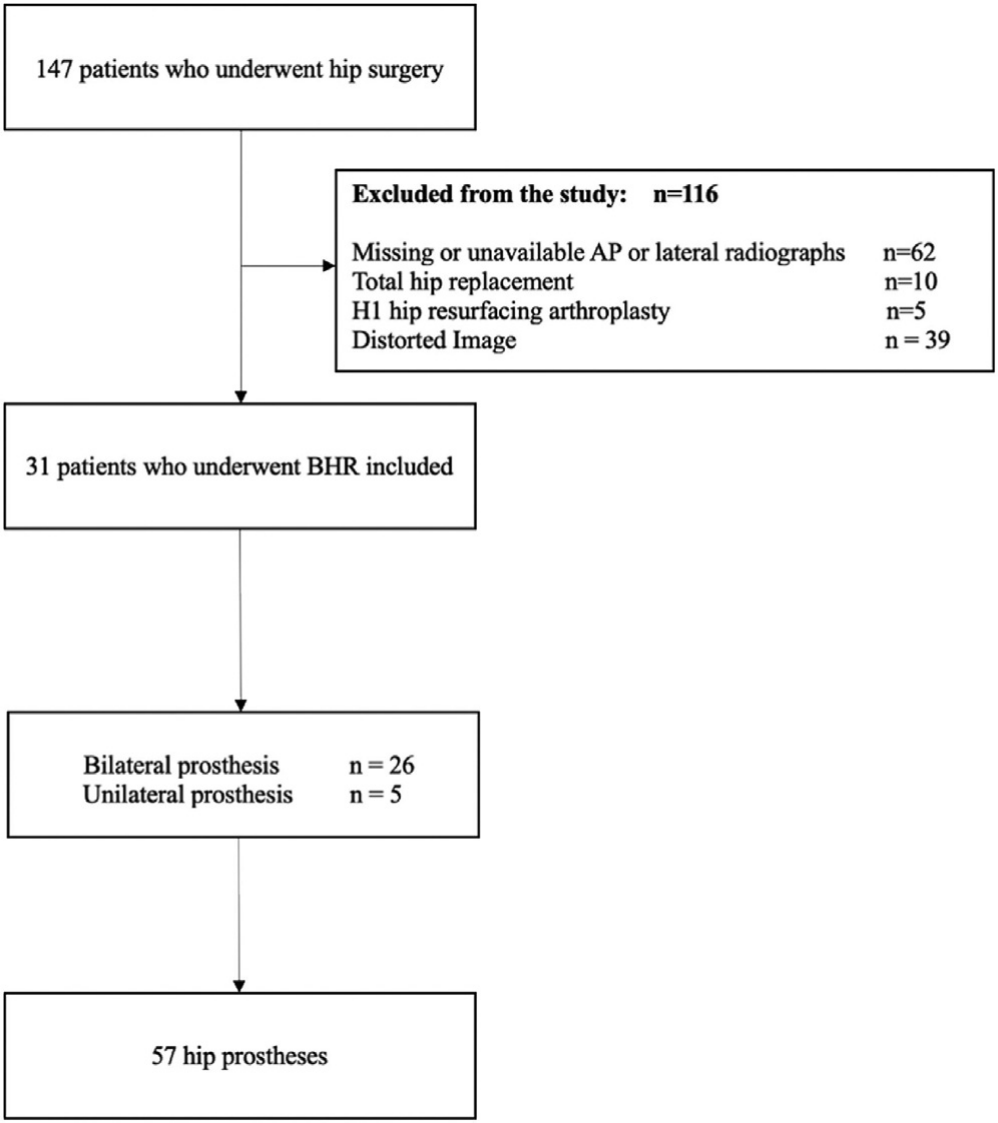
Patient selection.

Of the 147 patients identified, all of which had undergone hip surgery, 62 were excluded due to missing or unavailable AP or lateral radiographs. Ten patients were excluded as they had undergone THA. Five were excluded as they have undergone H1 HRA. Thirty nine patients were excluded due to inadequate imaging where the hip was incompletely visualized. The remaining 31 patients had undergone a BHR, of which 26 patients had bilateral prostheses, with the remaining 5 having a unilateral prosthesis. Overall, 57 hip prostheses were included. A flow summary of included and excluded patients is shown in Figure 4. For the fine tuning of the CV algorithm, 17 samples were selected (30%), and the remaining 40 samples were used to evaluate the performance of the model. Table 1 describes the characteristics of the included patients.

**Table 1:** Demographics table.

Characteristic	Count (n = 57)
Age (SD)	64.0 (10.2)
Sex (% female)	8.77
Years post operation (SD)	1.35 (1.05)

SD, standard deviation.

All values are given to 3 significant figures, per implant.

Figure 4 depicts the process of patient selection for this study, excluding patients without relevant and applicable imaging, or any history of any surgical intervention for the hip that was not BHS. Fifty seven total hips were included.

### Model performance

Table 2, Table 3 describe the outcomes of the 2 models for NSA calculation, showing that the final model significantly outperformed the baseline model across all performance metrics. The final model has a higher Spearman’s correlation (0.93 vs 0.83), a higher R^2^ (0.53 vs 0.37), and lower errors (mean absolute error: 1.94 vs 3.09 and MSE: 5.97 vs 19.3).

**Table 2:** Results of the 2 models compared with the ground truths to 3 significant figures.

Method	Mean	Standard deviation
AP: computer vision–generated NSA	135	7.92
AP: manual NSA	138	6.77
Lateral: computer vision–generated NSA	111	11.3
Lateral: manual NSA	N/A	N/A

AP, anterior-posterior; DNN, Deep Neural Network; NSA, neck-shaft angle.

Note that these results are before the use of the DNN.

**Table 3:** Results of the 2 models compared with the ground truths to 3 significant figures.

Method	r_s_ (*P* value)	R^2^	MAE	MSE
Without DNN and laterals	0.83 (<.01)	0.37	3.09	19.3
Final	0.93 (<.01)	0.53	1.94	5.97

DNN, Deep Neural Network; MAE, mean absolute error; MSE, mean square error.

The final model significantly outperformed the model that didn't include DNN and lateral radiograph data, with r_s_ of 0.93 and 0.83, respectively (*P* < .01). The final model depicted a better fit of the model to the data (R^2^ = 0.53, R^2^ = 0.37), with reduced errors.

## Discussion

In this study, we developed a novel CV model, using machine learning and CV, to detect hip prosthesis on radiographs, and to calculate the radiographic NSA, using a combination of AP and lateral radiographs. By using an edge detection algorithm, we were able to assess the radiomic NSA angles. The lateral and AP view data were used to train a DNN. The combined model, consisting of the DNN and both radiographic views, has a statistically significant correlation with the ground truth (r_s_ = 0.93, *P* < .01) with relative strength (R^2^ = 0.53) and little error (mean absolute error = 1.94, MSE = 5.97).

Given the evolution of deep learning models, from McCulloch-Pits Neuron Model of the neuron in 1943, to its current state, predicting and pioneering the next mainstream surgical application of deep learning is an area of great interest to the surgical field [26,27]. CV has been used in facial recognition, object detection, and human pose estimation [[28], [29], [30]], but few currently developed models use CV in a surgical context [[28], [29], [30]].

To the best of our knowledge, this is the first study that aims to develop a CV model to identify hip prosthesis and radiomic parameters on multiple radiographic views. A study by Choi et al. used a HIPPO algorithm to detect radiomic angles relating to hip dysplasia, including NSA, also known as caput-collum-diaphyseal angle, using AP pelvic radiographs, with high levels of reliability and speed [21].Yamada et al. have incorporated AP and lateral radiographic images to detect hip fractures, using Xception, a DNN model [31]. Yamada et al. demonstrated that their DNN model outperformed clinicians of varying experience levels in diagnosing femoral neck fractures, achieving recall, precision, and F1 scores of 1.00, 0.98, and 0.99, respectively [31].These results align with our study, further highlighting the effectiveness of AI-based models in analyzing multiple radiological views for accurate detection [31]. Similarly, the Xception model has demonstrated high accuracy (0.956) in identifying hip fractures on pelvic radiographs, aligning with the objectives of our research [32]. Additionally, Cheng et al. used TensorFlow and Urakawa et al. employed ResNet frameworks in their CV models, achieving accuracies of 0.91 and 0.955, respectively, in detecting various hip fractures [33,34]. These results were obtained using datasets from 3605 and 1776 patients, respectively, compared to the 31 patients in our study [33,34]. Moreover, Tanner et al.’s CV model used a YOLOv3-based deep learning algorithm to detect the hip-knee-ankle angle with high precision of 0.986 in THA and total knee arthroplasty patients, accurately identifying the femoral head [20]. However, their study relied solely on AP views, whereas our approach integrates multiple views for a more comprehensive and accurate assessment, underscoring the novelty of our proposed algorithm [20]. Despite the potential of CV in orthopaedic imaging and detection, there is a clear need for further model development [35,36].While existing studies tend to focus, individually, on the detection or classification of fractures, no studies to date have combined these approaches into a single model [37].

As a key indicator of proximal femur and hip joint geometry, the NSA has a key role in preoperative planning and surgical management of hip pathology [[38], [39], [40]]. The NSA is a technique used on AP radiographs. However, discrepancies in implant orientation within 3D space can introduce small errors in angle calculation. Lateral radiographs complement AP views by offering a more comprehensive assessment of prosthesis alignment and better contextualizing implant positioning, which AP imaging alone may not fully capture [[40], [41], [42]]. Incorporating both AP and lateral NSA calculations likely enhances anatomical accuracy by mitigating errors from rotational variability and improper limb positioning [[40], [41], [42]]. Clinically, lateral NSA measurements could enhance the accuracy of prosthesis positioning, reducing the risk of implant malalignment and mitigating the risk of future revisions [43]. Furthermore, enhanced preoperative planning using AI-driven NSA assessment could improve prosthesis selection and surgical approach, ensuring optimal outcomes for HRA procedures. As such, our algorithm offers an avenue for automating NSA measurement, reducing the margin of error by incorporating multiple views, thereby accurately quantifying the technical success of HRAs and informing future perioperative strategies.

To integrate our model into clinical practice within the National Health Service in the post-Brexit landscape, compliance with the United Kingdom Conformity Assessed marking, which has replaced the Conformitee Europeene marking under the Medical Devices Directive in the United Kingdom, is necessary to ensure safety, efficacy, and legal acceptance [44,45]. Adherence to Medical Device Regulations and Medicines Healthcare Regulatory Agency guidelines is also crucial for validating AI-based medical applications [46,47]. Furthermore, patient consent and transparency are critical in the ethical deployment of AI models, such as the one proposed in this study, into healthcare systems [48]. Patients should be informed when AI assists in surgical planning, ensuring transparency about its role, limitations, and reliability [49]. Clear documentation of AI-generated NSAs, and the overseeing clinician, would remain vital to uphold ethical standards in orthopaedic applications of AI [48,50]. While regulatory compliance may slow widespread adoption, it is necessary to ensure patient safety and clinical efficacy, ultimately supporting long-term integration into surgical workflows.

Our findings offer a novel insight into the use of CV in HRA. First, our model’s ability to identify HRA prostheses with a MSE CV algorithm has not been previously achieved. Second, by using an edge detection algorithm, a unique method of quantifying the angles in the radiographic images was employed. When this was used to train the DNN, we leveraged the inherent strengths of DNN for image analysis, incorporating AP and lateral radiographic views. Given the importance of preoperative planning, this offers surgeons a greater degree of accuracy for planning and classification, improving patient outcomes and operative success [51].

This study has several limitations. The sample size was small. Patients with missing lateral radiographs were excluded to maintain dataset consistency and model integrity, as the DNN relied on both AP and lateral NSA measurements for accuracy. Including patients with only AP scans could introduce heterogeneity, compromising predictive performance and direct comparisons with manual NSA values. Given the lack of multicenter external validation, there is a potential bias related to the generalizability of our results. Variation in radiographic protocols and practices across different institutions could impact the performance of the model in more diverse settings. Potential bias introduced by software or user interface is minimal, as all analyses were consistently conducted by our team using standardized software. While area under the curve, sensitivity, specificity, and accuracy are common reporting outcomes, our use of r_s_ and R^2^ were based on this study’s aim as a feasibility assessment, using these parameters to assess how random the outcomes of the CV model were, allowing for analysis of the model’s performance [33,35]. Finally, the model developed in this study is specifically designed for detecting hip resurfacing prostheses and calculating the NSA in postoperative radiographs. As such, it would require significant modifications to be adapted for preoperative templating of native hips. Therefore, this potential application was not included in our manuscript.

This study serves as a proof of concept, and therefore future studies can build on this work. Future research is required to validate this model using a larger, multicenter dataset with diverse demographics and postoperative timeframes, enabling more generalizable algorithms. An exploration into the effects of the NSA and radiomic factors surrounding hip prosthesis could be explored. Additionally, further CV models could be developed, for other imaging modalities, such as CT and MRI, could be developed. These modalities provide 3D anatomical details, reducing variability caused by patient positioning and projection distortions in standard radiographs [40,41]. Integrating CT or MRI data could refine NSA calculations by offering higher-resolution bone and soft tissue visualization. This could be achieved by training a proposed model on a multimodal dataset using deep learning architectures capable of processing volumetric data, such as the DNN used in this study, adapted for 3D imaging. This could then be applied to other joints. Moreover, analyzing the relationship between NSA post-HRA and postoperative outcomes, such as gait and function, could quantify the clinical relevance of NSAs within HRAs.

## Conclusions

Our study demonstrates the potential of using CV and DNN to identify hip prosthesis and NSA on radiographs. The robust performance of the developed model suggests its utility in clinical decision-making, potentially suggesting integration of CV into preoperative planning. Future research should focus on the relationship between gait and NSA and external validation of this model.

## Ethics approval

Ethical approval was received by NIES Committee North of Scotland IEC and the Imperial College Joint research compliance office (R&D reference number: 14HH2204).

## Data availability

Data on patients’ gait, imaging, and radiomics were collected, with patients consenting for all data to be used for future research.

An audio PaperClip is available at https://doi.org/10.1016/j.artd.2025.101717#mmc2.

## Funding

O. M. is sponsored by NIHR grant (NIHR ID 302632). J. P. C. is supported by funding from the Michael Uren Foundation Trust.

## Conflicts of interest

Justin P. Cobb received royalties from MatOrtho and Embody Orthopaedic; received speakers bureau/paid presentations for Zimmer Biomet and CeramTec; is the Director of Embody Orthopaedic Ltd.; is a paid consultant for JRI, DePuy, and Zimmer Biomet; holds stock or stock options in Embody Orthopaedic, Orthonika, and Additive Instruments; received research support from JRI Ltd., DePuy, Smith & Nephew, and Zimmer Biomet as a Principal Investigator; and reports institutional funding from the Sir Michael Uren Foundation. All other authors declare no potential conflicts of interest.

For full disclosure statements refer to https://doi.org/10.1016/j.artd.2025.101717.

## CRediT authorship contribution statement

**Omar Musbahi:** Writing – review & editing, Writing – original draft, Visualization, Software, Methodology, Investigation, Funding acquisition, Formal analysis, Conceptualization. **Savvas Hadjixenophontos:** Writing – review & editing, Methodology, Investigation, Formal analysis. **Saran S. Gill:** Writing – review & editing, Writing – original draft, Visualization, Formal analysis. **Iris Soteriou:** Writing – review & editing, Methodology, Formal analysis. **Kyriacos Pouris:** Writing – review & editing, Methodology, Investigation, Formal analysis. **Takuro Ueno:** Writing – review & editing, Writing – original draft, Methodology, Investigation. **Justin P. Cobb:** Writing – review & editing, Writing – original draft, Supervision, Resources, Methodology, Conceptualization.

## Appendix A Supplementary data

Conflict of Interest Statement for Soteriou

Conflict of Interest Statement for Cobb

Conflict of Interest Statement for Pouris

Conflict of Interest Statement for Musbahi

Conflict of Interest Statement for Hadjixenophontos

Conflict of Interest Statement for Gill

Conflict of Interest Statement for Ueno
