# Supplementary material for: Artificial Intelligence Approach in Hip Prosthesis Identification and Addressing Radiographic Outcome Measures
Source: Arthroplast Today. 2025 Jun 3;33:101717. doi: 10.1016/j.artd.2025.101717 (PMC12167101; doi:10.1016/j.artd.2025.101717)
Supplement: Conflict of Interest Statement for Cobb [file mmc2.docx]

# CONFLICT OF INTEREST STATEMENT

***American Association of Hip and Knee Surgeons***

(Adopted from the American Academy of Orthopaedic Surgeons disclosure statement)

The following form **must be filled out completely and submitted by each author (example, 6 authors, 6 forms).**

**All items require a response. If there is no relevant disclosure for a given item, enter "*None*.”**

**Manuscript Title: Artificial Intelligence approach in hip prosthesis identification and addressing radiographic outcome measures**

1. Royalties from a company or supplier (The following conflicts were disclosed)

Matortho, Emoby Orthopaedic

2. Speakers bureau/paid presentations for a company or supplier (The following conflicts were disclosed)

Zimmer Biomet, Ceramtec

3A. Paid employee for a company or supplier (The following conflicts were disclosed)

Director of Embody Orthopeadic Limited.

3B. Paid consultant for a company or supplier (The following conflicts were disclosed)

JRI, Depuy, ZimmerBiomet

3C. Unpaid consultants for a company or supplier (The following conflicts were disclosed)

None

4. Stock or stock options in a company or supplier (The following conflicts were disclosed)

Embody Orthopaedic, Orthonika, Additive Instruments

5. Research support from a company or supplier as a Principal Investigator (The following conflicts were disclosed)

JRI ltd, DePuy, Smith & Nephew, Zimmer Biomet

6. Other financial or material support from a company or supplier (The following conflicts were disclosed)

Justin Cobb reports institutional funding from the Sir Michael Uren Foundation

7. Royalties, financial or material support from publishers (The following conflicts were disclosed)

None

8. Medical/Orthopaedic publications editorial/governing board (The following conflicts were disclosed)

None

9. Board member/committee appointments for a society (The following conflicts were disclosed)

None

**Each author must sign AND print or type his/her name, date and submit a separate form**

In addition, one BLINDED Conflict of Interest form (no author names used) should be submitted per manuscript with all author disclosures.

Justin P Cobb JUSTIN P COBB 06.01.25

Author Name (Print or Type) Author Signature Date
